# Strengthening international patient advocacy perspectives on patient involvement in HTA within the HTAi Patient and Citizen Involvement Interest Group – Commentary

**DOI:** 10.1186/s40900-016-0053-8

**Published:** 2017-01-10

**Authors:** Janet L. Wale, Anna Mae Scott, Neil Bertelsen, Nick Meade, on behalf of HTAi Patient and Citizen Involvement in HTA Interest Group (PCIG)

**Affiliations:** 1 Patient advocate, 11A Lydia Street, Brunswick, Victoria 3056 Australia; 2 Centre for Research in Evidence Based Practice, Bond University, Gold Coast, Queensland 4229 Australia; 3 Neil Bertelsen Consulting, Eisenacher Straße 3, 10777 Berlin, Germany; 4 Genetic Alliance UK, Unit 4D Leroy House, 436 Essex Rd, London, N1 3QP UK; 5 Health Technology Assessment international (HTAi), 1200 10405 Jasper Avenue, Edmonton, Alberta Canada T5J 3N4

**Keywords:** Patient involvement, Patient engagement, Patient and citizen involvement interest group, Health technology assessment, Co-production, Evaluation

## Abstract

**Plain Language Summary:**

Health Technology Assessment (HTA) is an evidence-based decision-making process, focusing on evaluating health technologies for funding within a healthcare system. ‘Health technologies’ include medications, medical devices, diagnostics, medical procedures and services. Health Technology Assessment international (HTAi) is a global society for those who produce, use, participate in, or encounter HTA. The HTAi Secretariat supports special interest groups within HTAi. One such special interest group, the Patient and Citizen Involvement in HTA Interest Group (PCIG), focuses on strengthening patient involvement in HTA. PCIG’s members are from HTA agencies, research, industry, and patient organisations. We describe the steps PCIG has taken to form an international panel of patient advocates (the Patient Panel) as an autonomous group within its governance structure that reports directly to the PCIG Steering Committee. The Patient Panel was established in order to strengthen meaningful patient involvement in HTA by working co-productively with other PCIG members to develop resources accessible to patient organisations.

Patient advocates known to be active within HTA in their own countries were invited to form the inaugural group, with one person appointed to chair the group for the first year. Documentation had been prepared to inform potential members and set out potential roles as seen by the Steering Committee. The appointed Patient Panel is defining its own terms of reference, goals and objectives. The Panel Chair is a member of the PCIG Steering Committee, and is mentored by the retiring PCIG chair. A registry of activities is part of monitoring and evaluation.

**Abstract:**

**Background**

Health Technology Assessment (HTA) is an evidence-based decision-making process, focusing on evaluating health technologies for funding within a healthcare system or medical insurance system. ‘Health technologies’ are understood broadly, and include medications, medical devices, diagnostics, medical procedures and services. Health Technology Assessment international (HTAi) is a global society for those who produce, use, participate in, or encounter HTA. The HTAi Secretariat supports special interest groups within HTAi, including the Patient and Citizen Involvement in HTA Interest Group (PCIG), which focuses on strengthening patient involvement in HTA. PCIG’s members come from HTA agencies, research, industry, and patient organisations.

**Main body**

We describe the steps the PCIG has taken to form an international panel of patient advocates as an autonomous group within its governance structure, reporting directly to the PCIG Steering Committee. Patient advocates known to be active within HTA in their own countries were invited to form the inaugural group. One person was invited to chair the Panel for the first year of its operation, based on the candidate’s experience of working across diseases and countries, and being new to HTAi. Documentation was prepared to inform potential members of the Panel and set out potential roles as seen by the Steering Committee. The Panel came into being in March 2016 and is now setting out its own working principles, goals and objectives. The Panel Chair is a member of the PCIG Steering Committee, and is mentored by the previous PCIG chair. A registry of activities has been set up as part of monitoring and evaluation.

The Patient Panel is intended to provide a focus within the PCIG for patient advocates to work among themselves and co-productively through the PCIG Working Groups to develop mutual understanding and strengthen meaningful patient involvement in HTA internationally. Patient advocates can benefit from a clear understanding the evidence requirements within HTA and how information can be effectively presented by patient groups to decision-making bodies.

**Conclusion**

The HTAi Patient and Citizen Involvement Interest Group has set up a Patient Panel consisting of patient advocates. The intent is to work co-productively to advance patient involvement in HTA.

## Background

Health Technology Assessment (HTA) is an evidence-based decision-making process, focusing on evaluating health technologies for funding within a healthcare system or medical insurance system. HTA is formally defined as “the systematic evaluation of the clinical effectiveness and/or cost-effectiveness and/or the social and ethical impact of a health technology on the lives of patients and the health care system” [1]. ‘Health technologies’ are understood broadly, and include medications, medical devices, diagnostics, medical procedures and services.

Health Technology Assessment international, or HTAi, is the international society for the promotion of HTA as a means of informing evidence-based healthcare decision making. HTAi’s mission is to support and promote the development, communication, understanding and use of HTA around the world as a scientifically-based means of promoting the introduction of effective innovations and the effective use of resources in health care [2]. HTAi also serves as an umbrella organisation for interest groups focused on specific aspects of HTA, such as ethics, hospital-based HTA, HTA in developing countries, and patient and citizen involvement [3].

The Patient and Citizen Involvement in HTA Interest Group (PCIG) is the focus of the present commentary, in particular the process and outcome of PCIG’s initiative to set up a Patient Panel within its governance structure. By sharing our process and its outcome we aim both to attract interest in the Panel and to assist other groups endeavouring to establish similar panels.

HTAi collects annual membership dues from its members, and organises annual meetings for which it seeks both public and industry sponsorship. Reduced membership fees are paid by patient advocates, although at present it is possible to join the PCIG without becoming an HTAi member. In order to encourage participation in the annual meetings, patient advocates and delegates from low and low-middle income countries have the opportunity to apply for travel grants, made available by HTAi. Some funding is also annually distributed by HTAi among the interest groups, including PCIG. In recent years, PCIG has sought funding from the HTAi Board to enable a yearly face-to-face meeting to further its work; this covers at least some of the participants’ expenses. Other funding for PCIG’s activities has come from grants from industry for specific projects, and in 2016 from the Pharmaceutical Research and Manufacturers of America (PhRMA) through the HTAi Secretariat. However, the bulk of the work within the PCIG, including as members of PCIG working groups and the PCIG Steering Committee, is carried out in members’ own time and is unpaid by HTAi. The interest groups provide an annual report to the HTAi Board, including details on expenditures. The chairs of the interest groups are part of an HTAi Board-led steering committee as a mechanism to provide accountability on the part of the interest groups and the Board.

## Main text

### The Patient and Citizen Involvement in HTA Interest Group

The Patient and Citizen Involvement in HTA Interest Group (PCIG) was established in 2005. The overarching aim of the PCIG is to enhance the quality and relevance of HTA through patient and citizen involvement [4]. The PCIG objectives are to: either promote or develop robust methodologies to incorporate patient perspectives in HTA, and share good practices in patient and public involvement in HTA processes; strengthen HTA by systematic incorporation of patient perspectives; support countries with limited experience with HTA and incorporating patient and citizen perspectives; and demonstrate the value and impact of patient and public engagement in HTA.

PCIG’s membership consists of over 250 individuals from HTA agencies, industry, patient advocates, researchers and others who are interested in or working with patients and caregivers who seek access to healthcare interventions through formal mechanisms such as HTA. Members are linked through a monthly e-bulletin, and have the opportunity to convene at an annual business meeting held during HTAi annual meetings, as well as to participate in workshops and panel sessions held at this event.

**Fig. 1:**
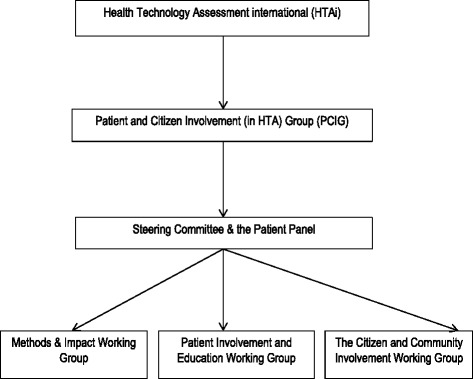
Organisational structure of the HTAi Patient and Citizen Involvement in HTA Interest Group (PCIG)

PCIG is organised into three working groups (see Fig. 1) and members can volunteer to participate in a working group that is aligned to their particular interests. The objectives of the Methods and Impact Working Group are to identify robust evidence that can impact on HTA decision making about the views and preferences of patients. It aims to share information about research methodologies and research findings and build an evidence base for the impact of patient involvement in HTA. The Patient Involvement and Education Working Group seeks to promote knowledge, skills and opportunities for meaningful patient involvement in HTA activities in countries in all regions of the world. The Citizen and Community Involvement Working Group concentrates on the role of public or citizen and community involvement in HTA. The members of all three working groups collaborate on projects both within and across working groups, develop materials and resources, and identify gaps in current knowledge around patient and citizen involvement in HTA. Some of these projects have included: the Values and Quality Standards for patient involvement in HTA, generic templates for patient group submissions to HTA agencies, guidance on making submissions, ethics guidance for patient organisations, and frequently asked questions [4, 5].

The structure and work of the PCIG is overseen by a Steering Committee. The Steering Committee’s remit is to oversee the governance and activities of the PCIG, including coordination of the overall strategy, promotion of the outputs of the Interest Group, and reporting back to the HTAi Board through the Chair. The Steering Committee consists of 12 members who are researchers, people working with patient and public involvement in HTA agencies, independent consultants, a patient organisation (European Patients’ Forum) representative, and patient advocates. It was the Steering Committee that saw the need to strengthen the voices of patient advocates in the work of the PCIG by forming an international group of patient advocates with experience in HTA. This could be done in a focused and collaborative way by following the concepts of person-centred health care, in that a health system is there to meet the needs of patients.

### Principles followed by the Patient and Citizen Involvement in HTA Interest Group

As part of its work, the PCIG has developed a set of values or principles to be upheld in the involvement of patients and the public in HTA processes, together with quality standards [6]. The values are as follows:

- Relevance: Patients have knowledge, perspectives and experiences that are unique and contribute to essential evidence for HTA.
- Fairness: Patients have the same rights to contribute as other stakeholders and have access to processes that enable effective engagement.
- Equity: Patient involvement contributes to equity by seeking to understand the diverse needs of patients with a particular health issue, balanced against the requirements of a health system that seeks to distribute resources fairly.
- Legitimacy: Patient involvement facilitates those affected by the HTA recommendations or decisions to participate; contributing to the transparency, accountability and credibility of the decision-making process.
- Capacity building: Patient involvement processes address barriers to involving patients and build capacity for patients and HTA organizations to work together.

These values are directed at HTA agencies and have been acknowledged, referenced and where applicable translated by HTA agencies in England, Scotland, France, Belgium, Colombia and Canada [7]. They are also applicable to the Patient Panel and underpin its ability to function effectively within the PCIG.

### Patient-centeredness

In May 2014, the World Health Organization approved a resolution urging member states to establish and utilise health technology assessment processes in support of universal health coverage [8]. Inclusion of patient perspectives in the HTA process can therefore be seen as a means of supporting and ensuring universal access to healthcare interventions that are needed, appropriate, and accepted by patients for their care [8].

Some countries have directed their efforts toward patient-focused HTA, in line with the principles of person-centred health care that advocate shared decision making, self-management, and patient empowerment [9, 10]. England, Scotland, and Canada, for example, have established mechanisms for the involvement of patients and the public in their HTA processes, at The National Institute for Health and Care Excellence (NICE), Scottish Medicines Consortium (SMC), and the Canadian Agency for Drugs and Technologies in Health (CADTH), respectively.

In line with health systems that embrace patient involvement in health care and health policy, as well as with its own set of principles, the PCIG has restructured to be more inclusive of patient advocates and to set up a Patient Panel. The Panel is intended to provide patient advocates and their patient organisations a stronger voice in the governance and activities of the Interest Group.

### Setting up the HTAi Patient Panel

The possibility of establishing an international group of patient advocates within the PCIG was first raised in 2015, by the then Chair (JW). This concept was raised with the PCIG Steering Committee (SC) as a means of achieving a more democratic and effective way of operating the PCIG. It was modelled on the advisory group of regulators that has been set up to provide regulatory support to another interest group within HTAi, the HTA-Regulatory Interactions Interest Group. The model of a multi-stakeholder patient involvement interest group that incorporates a definable group of patient advocates to give them a well-defined voice was therefore adopted from within HTAi itself, rather than from other international organisations in HTA. It differs from the International Society for Pharmacoeconomics and Outcomes Research (ISPOR) model of a Patient Centred Special Interest Group and its Patient Engagement in Research Working Group that involves patient advocates and other HTA stakeholders in clinical outcomes research [11, 12]. The Steering Committee recognised the advantage of having a defined group of patient advocates working within the PCIG and therefore making valuable contributions to the work of the PCIG, following the principle of ‘together we are stronger’.

It was necessary to prepare documents for potential Panel members, with information on HTA, HTAi, the PCIG itself, as well as on the envisioned roles for the potential members of the Patient Panel [13].

The Chair of the Patient Panel was to become a member of the PCIG SC; and the Patient Panel was to sit alongside the SC (see Fig. 1). Establishing the Patient Panel required revision of the Terms of Reference of the SC, as well as a strengthening of the process for declaration of interests. The latter was done to improve transparency about membership of the Interest Group. These changes were approved by the Interest Group members at its 2016 Annual Business Meeting.

Identification of potential members of the Patient Panel was undertaken by the then Chair, herself a patient advocate, and the other Steering Committee members. PCIG were looking for a balanced group of patient advocates in the area of HTA who were held in high esteem by their colleagues, and international representation. One of the considerations around asking patient advocates to become involved is that they are busy individuals with high demands on their time, and HTAi is not the only international not-for-profit organisation working in this space – others include, for example, ISPOR and Develop Innovate Advance (DIA), which are also actively involved in the European public-private Innovative Medicines Initiative (IMI) funded European Patients Academy on Therapeutic Innovation (EUPATI). However, the PCIG has shown itself to be effective as a group in working with patient advocates and others to develop a library of resources that are widely used, as previously described. We were able to send out formal invitations to potential Panel members, and the Patient Panel was finalised in March 2016, with members of the PCIG formally notified in the same month. The Chair of the Patient Panel was identified after careful consideration of the composition of the Panel. The key considerations were that: the candidate be knowledgeable in the area of HTA, has experience in working with people from patient support groups across a range of diseases and from different countries, and that they were new to the PCIG. The Patient Panel Chair is being mentored by the now previous chair of the PCIG, and provided with secretarial support, to ensure that they are supported as they take up this collaborative role. Although the inaugural Chair of the Patient Panel was identified by the SC, and appointed for an initial period of one year, it is envisioned that the Panel will subsequently appoint its own chair. The Chair is a member of the Steering Committee of the PCIG under the new PCIG SC Terms of Reference.

Already the international Patient Panel is becoming familiar with the work of the HTAi, PCIG and their members, and is exploring ways in which its efforts can be mutually beneficial. The first face-to-face interactions took place at the HTAi annual conference in June 2016, in Tokyo, where the Patient Panel actively contributed by commenting on the clarity of the website for the event in terms of who the HTAi stakeholders actually are; providing background material [14]; presenting at the PCIG full-day workshop ‘East Meets West: what we can learn from each other about patient perspectives and adding value to HTA and policy decision making’; providing input into a Policy Forum Panel presentation; and attending the 2016 Annual Business Meeting. It also contributed to the 2016 PCIG Working Groups’ annual face-to-face meeting. Panel members are particularly interested in learning from each other and from the PCIG about how patient involvement in HTA in their own countries can be built on and improved, and upskilling in cross-border collaboration on HTA, for example in the European Union.

### Identified roles of the Patient Panel

The Patient Panel is intended to be a collaborative and supportive ‘forum’ of patient advocates, developing discussions to address the purpose, processes, facilitators and barriers for patient and public involvement in a range of countries. To this end, it is envisioned that the Patient Panel members will collaborate with other PCIG members to strengthen methodologies for capturing patient perspectives in HTA. They will develop their own goals and objectives. Until then, the Patient Panel’s key roles, as identified by the SC, include:

- providing a strong representation of patient views alongside those of the views of other stakeholders within the PCIG;
- enabling a supportive environment for patient advocates as valued stakeholders;
- acting as a sounding board for new PCIG projects and contributing ideas, assessing their relevance and practicality for patient organisations;
- developing their own projects through broad discussions.

It needs to be emphasised that the Patient Panel will devise its own work plan, priorities and terms of reference. A registry of activities has been initiated to facilitate an evaluation of their activities within the PCIG.

### Patient Panel membership

Currently, a core group of nine international patient advocates with experience in contributing to HTA processes in their own countries form the Patient Panel. This number is anticipated to grow as the Panel becomes more established. The current members come from one international organisation and six national organisations, in England, Scotland, Netherlands, Canada, Taiwan, and Australia. Because the Panel is self-governing, it can broaden its membership to add strengths and to encompass more countries.

### Moving forward

The impetus for forming the Patient Panel came from within the HTAi PCIG Steering Committee, who sought patient advocates with HTA experience, the potential to make a valuable contribution to the Panel, and who were willing and committed to be part of the Panel. International diversity was a key consideration. The SC designed a structural framework for the Patient Panel while leaving details on how it would operate and the principles under which it would function up to the Panel and its Chair to develop, with approval by the SC. The Patient Panel have had one teleconference, in September 2016, to begin discussions on these.

Some of the Patient Panel members attended the HTAi Annual Meeting in May 2016 and contributed to the PCIG one-day workshop. The Chair and two of the Patient Panel were able to attend the face-to-face meeting of the PCIG Working Groups in October 2016. Members who were present confirmed that the Panel is needed within the PCIG, as a source of patient opinions. Members value being involved in Working Group discussions as well as having the opportunity to have their own discussions and to share experiences. Each member belongs to a patient organisation and has access to other patient groups, so has the capacity to consult widely. The Patient Panel Chair stated that membership is based on the skills of the group (rather than country ‘representation’). It is important that they are accessible to each other and the PCIG, and that they feel empowered to provide input, even if not asked. The terminology is often unclear in HTA such that a common language is needed for communication and upskilling relevant to HTA. Many patients and patient organisations are not aware of HTA and its function. HTA also needs to be transparent and patient input and its impact made visible. Conflicts of interest need to be managed. The Patient Panel will be part of the solution to these issues by working collaboratively with PCIG and HTAi.

The Patient Panel needs to identify best strategies for communicating with the PCIG and, at the same time, how to be of most value to the patient community. It can also undertake to ﻿lead on better relationships between patient organisations and HTA agencies, outside of the formal relationships within agency advisory groups.

### Challenges

The Patient Panel, as with the PCIG, has members from different countries. As with many international organisations, English is the language spoken within HTAi but is a second language for many people, which may present communication challenges. The structure of health systems and their culture also vary globally, and the role of patient advocates within the health systems. The PCIG has members from different stakeholder groups within HTA – including industry, researchers, decisions-makers, patients – who may have different perspectives on patient involvement in HTA. Furthermore, the PCIG, including the Patient Panel, has a limited budget, relying largely on goodwill to achieve its goals. Funding limits the ability to attend face-to-face meetings and much of its work is done through e-mail and teleconferencing using facilities provided by the HTAi Secretariat. However, face-to-face meetings occur at the HTAi Annual Meetings and in the second quarter of the year for Working Groups and now the Patient Panel.

## Conclusions

We describe here the initiative, the process, and the outcome of establishing a Patient Panel within the HTAi Patient and Citizen Involvement in HTA Interest Group. Although the Panel is still in its early stages, it will considerably strengthen and enrich the work of the PCIG, in addition to being consistent with the PCIG’s own values and the increasing emphasis on patient-centeredness in health systems around the world.

## Abbreviations

CADTH: Canadian Agency for Drugs and Technologies in Health
DIA: Develop Innovate Advance
EUPATI: European Patients Academy on Therapeutic Innovation
HTA: Health technology assessment
HTAi: Health Technology Assessment international
IMI: Innovative Medicines Initiative
ISPOR: Society for Pharmacoeconomics and Outcomes Research
NICE: The National Institute for Health and Care Excellence
PCIG: HTAi Patient and Citizen Involvement in HTA Interest Group
SC: PCIG Steering Committee
SMC: Scottish Medicines Consortium
